# Case Report: Abscopal effect of radiotherapy in a patient with metastatic duodenal adenocarcinoma and resistance to chemoimmunotherapy

**DOI:** 10.3389/fimmu.2025.1643197

**Published:** 2025-12-16

**Authors:** Yongping Hao, Jin Wang, Yinling Wang, Zihui Wang, Guangwei Tian, Nan Li

**Affiliations:** 1 Department of Radiation Oncology, the First Affiliated Hospital of China Medical University, Shenyang, China; 2 Department of Medical Oncology, the First Affiliated Hospital of China Medical University, Shenyang, China; 3 National Clinical Research Center for Laboratory, the First Affiliated Hospital of China Medical University, Shenyang, China; 4 Department of Anesthesiology, University of Maryland School of Medicine, Baltimore, MD, United States

**Keywords:** abscopal effect, chemotherapy resistance, duodenal adenocarcinoma, immunotherapy resistance, metastatic disease, radiotherapy

## Abstract

**Background:**

Duodenal adenocarcinoma is a rare gastrointestinal malignancy, with limited clinical treatment data and a poor prognosis, especially when metastatic. Standard therapies such as chemotherapy and immunotherapy often exhibit limited efficacy, particularly in advanced stages of the disease. The abscopal effect, where local radiotherapy leads to regression of distant metastatic lesions, has been documented in various cancers but is not well explored in duodenal adenocarcinoma. This case report evaluates the potential for radiotherapy to induce an abscopal effect in a patient with metastatic duodenal adenocarcinoma resistant to chemotherapy and immunotherapy.

**Objective:**

To explore the potential of radiotherapy to induce an abscopal effect and provide palliative benefits in a patient with advanced, treatment-resistant metastatic duodenal adenocarcinoma.

**Results:**

A 60-year-old female with metastatic duodenal adenocarcinoma, resistant to chemotherapy and immunotherapy, underwent palliative radiotherapy targeting eight metastatic sites. The patient received 40Gy in 20 fractions, completing 19 due to poor physical condition. Notably, radiotherapy resulted in significant regression of a metastatic lesion in the right maxillofacial region, which was not irradiated, demonstrating an abscopal effect. Tumor markers decreased, and the patient experienced substantial pain relief. Immune analysis revealed a decrease in granulocyte and lymphocyte counts and elevated IL-6 levels, which persisted throughout the treatment. Despite this positive response, the patient passed away after 23 months due to disease progression.

**Conclusion:**

This is the first reported case of an abscopal effect in metastatic duodenal adenocarcinoma, where radiotherapy not only controlled the primary lesion but also induced regression of distant metastatic lesions. These findings suggest that, for patients with multiple metastases, localized radiotherapy could be a feasible palliative option for pain relief and may provide short-term benefit. Larger studies are warranted to confirm this conclusion.

## Introduction

Malignant tumors of the small intestine are relatively uncommon. In 2023, approximately 12,070 new cases of small intestine carcinoma were reported in the United States, resulting in about 2,070 deaths (1). Adenocarcinomas constitute roughly 36.9% of these cases (2), with most small intestine adenocarcinomas originating in the duodenum (2, 3), particularly in the D2 segment (3, 4). Despite an increasing incidence in recent years (5), clinical treatment data for duodenal adenocarcinoma remain scarce. Consequently, it was often grouped with other periampullary tumors (6), with surgery being the primary therapeutic option. Compared to other periampullary adenocarcinomas, duodenal adenocarcinoma has a relatively better prognosis, with a 5-year tumor-specific survival rate of approximately 55-59% (7, 8). Prognostic factors for overall survival (OS) include tumor stage (TNM III), neural invasion, a lower platelet/lymphocyte ratio, and elevated CA19-9 (9, 10). Patients with pT4N2 duodenal adenocarcinoma have a prognosis similar to those with metastatic disease, with approximately 75% of patients experiencing distant metastasis within one year post-surgery (9).

Radiotherapy is a key treatment modality for cancer. The concept of the abscopal effect was first introduced by Mole in 1953, describing the phenomenon in which non-irradiated lesions also experience inhibition or shrinkage in addition to the irradiated tumor sites (11). Since 1969, numerous cases have documented the occurrence of the abscopal effect during treatment. Melanoma is the most commonly observed cancer associated with this effect (12–14), followed by hepatocellular carcinoma (15, 16), non-small cell lung cancer (17), and sarcoma (18). Beyond radiotherapy, local treatment approaches such as photodynamic therapy (19), sonodynamic therapy (20), photothermal therapy (21), microwave ablation (22), cryoablation (23) and cytoreductive surgery (24) have also been shown to induce the abscopal effect. The mechanisms underlying the abscopal effect remain unclear. Recent research suggests that factors such as the type of radiation, radiotherapy dose and fractionation (25), p53 status, immune status, tumor type, and the sequence of anti-tumor treatments may influence its occurrence (26). With the growing importance of immunotherapy in cancer treatment, an increasing number of abscopal effects have been reported in patients receiving combined radiotherapy and immunotherapy (17, 18, 27, 28).

In this report, we present a case of an abscopal effect induced by radiotherapy in a patient with advanced duodenal adenocarcinoma and multiple metastases, who was resistant to immunotherapy. We anticipate that this case will offer valuable insights into the treatment of patients with advanced duodenal cancer.

## Case presentation

In November 2022, an adult patient presented to a local hospital with unexplained jaundice of the skin and sclera. MR imaging (MRI) indicated a low-level biliary obstruction, and CT imaging revealed a mass in the duodenum (**Supplementary Figure 1**). On December 8, 2022, the patient underwent a pancreaticoduodenectomy and cholecystectomy. Intraoperatively, the tumor, located at the major papilla of the duodenum, measured approximately 1.5 × 1.5 × 1.0 cm. Postoperative pathology confirmed a moderately to poorly differentiated adenocarcinoma of the duodenal major papilla, involving the common bile duct, penetrating the muscular layer of the small intestine to reach the subserosa, but sparing the pancreas. Vascular tumor emboli were observed, with no abnormalities at the resection margins of the bile duct, jejunum, stomach, or pancreas. Lymph node metastasis was detected in the 12B and 12P groups, while the 12A, 12P, 13, 7, 8, and 9 lymph node groups were negative for cancer cells. Genetic testing revealed a KRAS exon 2 p.G12R (c.34G>C) mutation, a tumor mutation burden (TMB) of 4.93 mutations/Mb, and microsatellite stability (MSS). The tumor proportion score (TPS) for PD-L1 expression was <1%, and the combined positive score (CPS) was 3. Postoperatively, the patient presented to our hospital for comprehensive baseline evaluation. Serum tumor markers CEA and CA19–9 were both within normal limits, and contrast-enhanced thoracic and abdominal CT showed no evidence of metastatic disease. Taken together, these findings indicated a clinical stage of T3N1M0 (stage IIIA).

On January 17, 2023, the patient began treatment with one cycle of monotherapy using capecitabine, followed by a visit to the surgery department for the removal of the drainage tube. She continued with 3 cycles of chemotherapy using the CAPOX regimen (capecitabine and oxaliplatin). In April 2023, an Emission Computed Tomography (ECT) scan revealed increased metabolic lesions in the right maxilla, multiple sites in the spine, left ribs, right iliac wing, and the proximal right femur. MRI of the thoracic and lumbar spine confirmed abnormal enhancement in the left accessory area of T8, T9, L2, and within the body of L3 vertebra, indicating multiple bone metastases. The Response Evaluation Criteria in Solid Tumors (RECIST) version 1.1 evaluation showed progressive disease (PD). Consequently, the treatment regimen was modified on April 26, 2023, to include CAPOX combined with bevacizumab for 4 cycles. Due to significant side effects from oxaliplatin, the 5th cycle was administered with only capecitabine and bevacizumab.

**Figure 1:**
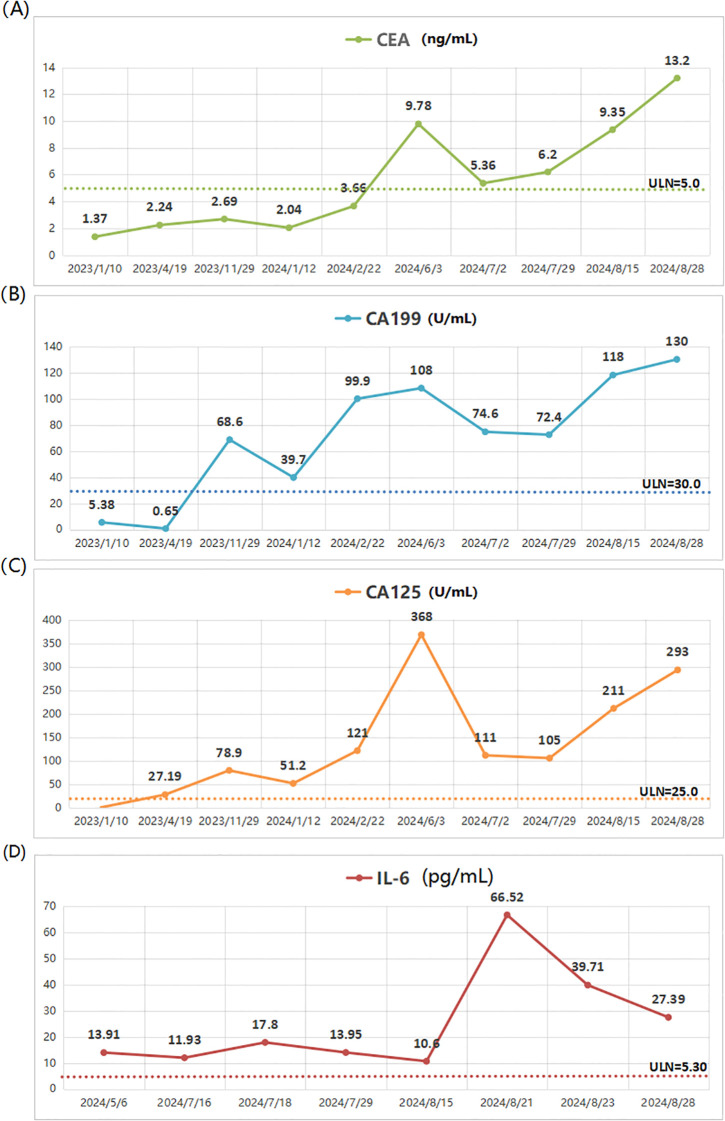
The changes in the patient’s tumor markers and cytokines during the treatment. **(A)** CEA. **(B)** CA-199. **(C)** CA-125. **(D)** IL-6.

In December 2023, a PET scan revealed multiple areas of increased bone density, with slightly lower density shadows in the bilateral gluteal muscles and the right thigh muscle, suggesting metastatic malignant lesions. Concurrently, tumor markers CA-199 and CA-125 increased to 68.6 U/mL and 78.9 U/mL, respectively(**Figure 1**). All examinations confirmed PD, and treatment was switched on December 21, 2023, to 2 cycles of chemotherapy with irinotecan and raltitrexed, combined with bevacizumab. Following this treatment, CA-199 and CA-125 levels decreased. In February 2024, the patient experienced pain in the right maxillofacial region, and enhanced CT scans suggested a dermoid cyst. The lesion in the right piriformis had increased in size compared to previous assessments, while tumor markers rose again, with CA-199 increasing to 99.9 U/mL and CA-125 increasing to 121 U/mL. Due to rapid disease progression, treatment was changed to a combination of liposomal irinotecan, toripalimab, S-1, and lenvatinib for a total of 2 cycles. However, after two cycles, the patient’s physical status deteriorated, with generalized pain and inability to tolerate chemotherapy. The patient was prescribed oral morphine and oxyContin for pain relief.

**Figure 2:**
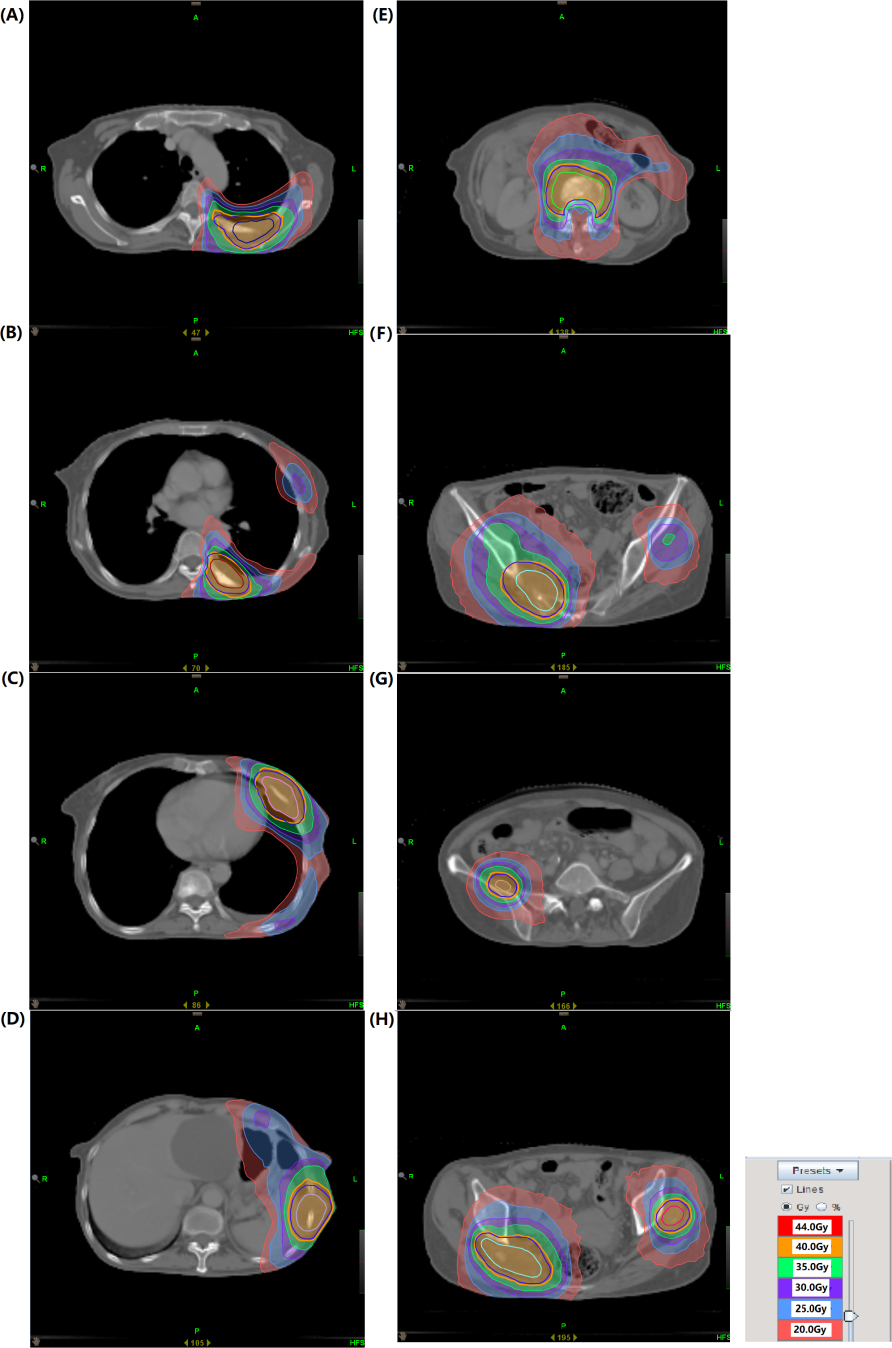
**(A–H)** Representative dose distributions of 8 lesions.

**Figure 3:**
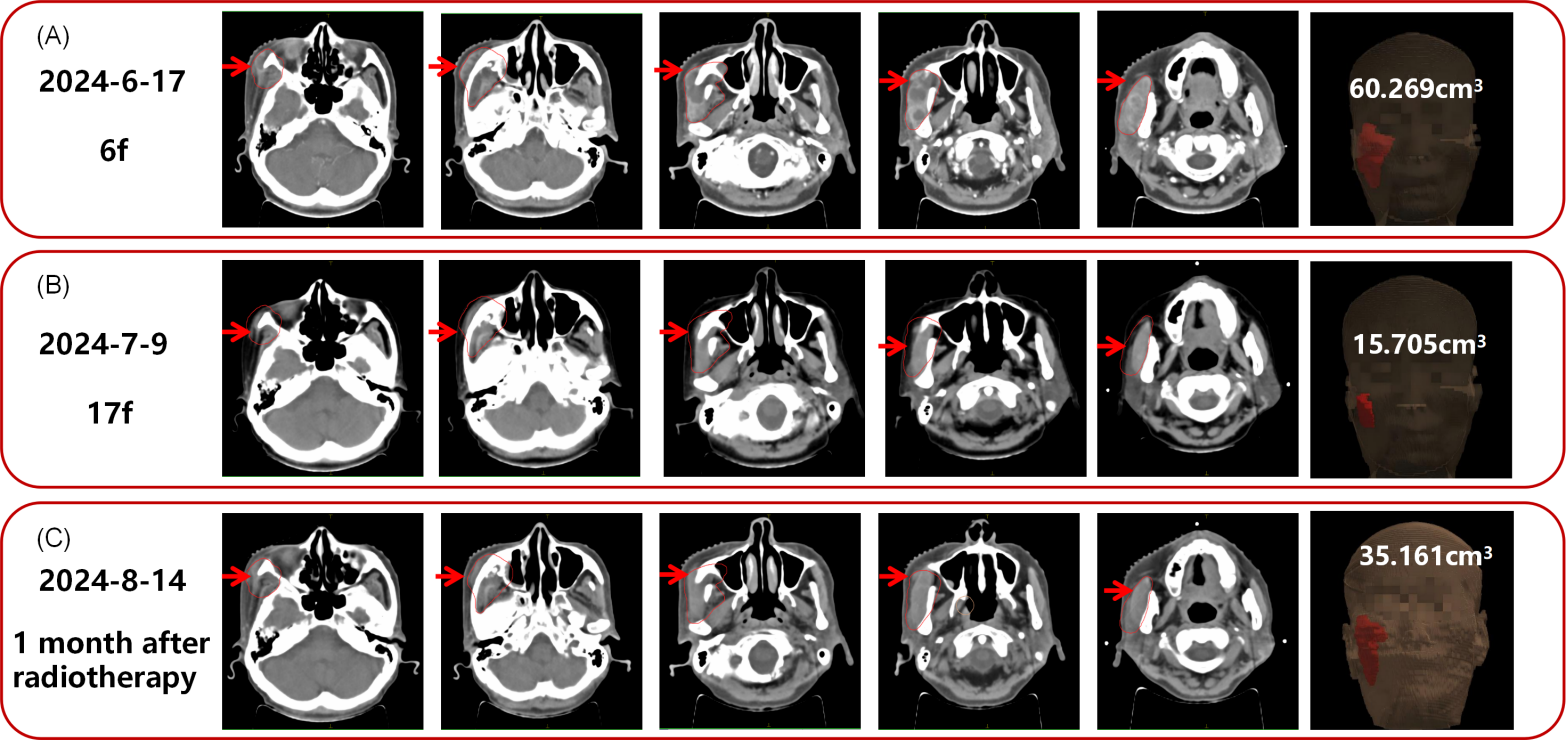
CT images of the patient’s maxillofacial region. **(A)** At the 6th radiotherapy fraction. **(B)** At the 17th radiotherapy fraction. **(C)** One month after the completion of radiotherapy.The red contour indicates the tumor delineation on June 17, 2024, serving as a baseline for comparison with subsequent tumor changes. The red arrow points to the tumor location, and the tumor volume is labeled on the last image of each time point.

In May 2024, due to severe pain in multiple regions, including the left chest, right buttock, and right maxillofacial area, the patient sought palliative pain-relief radiotherapy. Imaging demonstrated multiple osseous and soft-tissue metastases throughout the body. The eight most painful lesions were treated, including the left ribs, vertebrae, right ilium, right piriformis, and left gluteus minimus, from June 7 to July 18, 2024 (**Supplementary Figure 2**). The representative dose distribution is shown in **Figure 2**. Given the patient’s poor performance status, we prescribed 40 Gy in 20 fractions. This regimen yields a BED similar to that recommended by the ASTRO guideline (29) for bone-metastasis radiotherapy, with a lower dose per fraction and thus fewer side-effects. However, only 19 fractions were completed due to the patient’s poor physical condition. Notably, the patient’s pain was significantly alleviated during treatment, and the left rib lesion visibly shrank. Tumor markers also gradually decreased (**Figure 1**). Remarkably, the radiotherapy induced an abscopal effect. After only 3 treatments targeting body metastases, the lesion in the right maxillofacial region, which had not received radiotherapy, significantly shrank and continued to shrink in subsequent treatments. CT scans of the maxillofacial region at the 6th, 17th fractions regression of the metastatic lesions, attributable to the abscopal effect (**Figure 3**). The maxillofacial lesion volume decreased from 60.269 cm³ to 15.705 cm³.

During radiotherapy, the patient’s granulocyte count decreased to 0.67 × 10^9/L (**Supplementary Table 1**). After granulocyte-stimulating factor administration, radiotherapy continued. The patient’s lymphocyte levels were significantly affected; prior to treatment, the lymphocyte count was 1.34 × 10^9/L (normal range: 1.1 × 10^9/L to 3.2 × 10^9/L). After 5 fractions, the lymphocyte count dropped to 0.4 × 10^9/L and remained low throughout the treatment. At the end of radiotherapy, various T-cell counts decreased, with a particularly notable decline in the percentage of CD4+ T cells. Blood biochemistry and T-cell subset analysis results are presented in the supplementary materials (**Supplementary Tables 2**, **3**). Cytokine testing showed a significant elevation in IL-6 levels, which increased from 13.91 pg/mL before treatment and remained elevated during therapy (**Figure 1**). Other cytokines did not show significant abnormalities (**Supplementary Table 4**).

**Figure 4:**
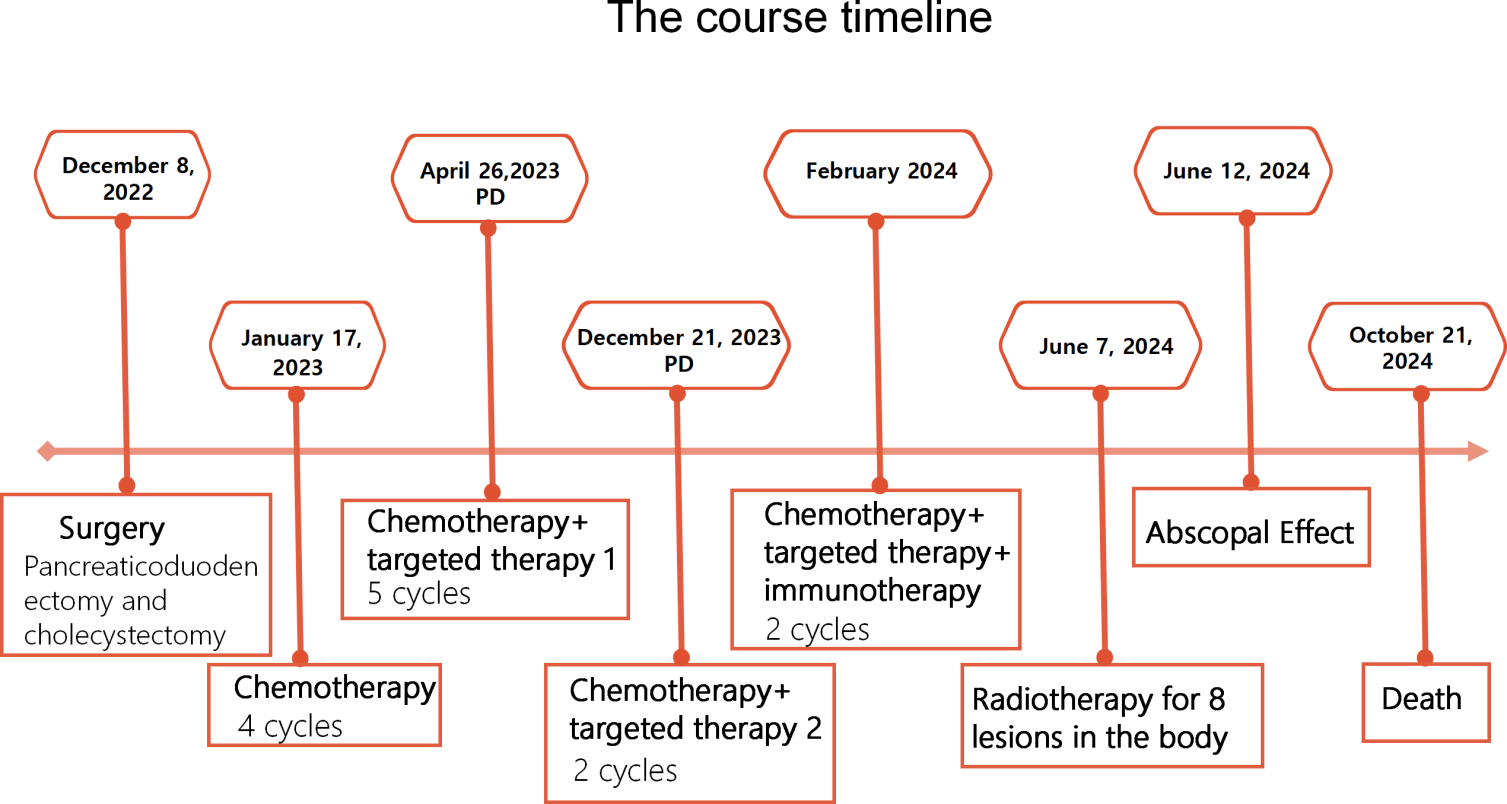
The course timeline illustrating treatments of the patient.

After completing radiotherapy,whole-body CT revealed enlargement of multiple other non-irradiated lesions, consistent with progressive disease. The unradiated systemic lesions progressed, yet the non-irradiated maxillofacial lesion regressed, indicating inter-lesional heterogeneity. The patient developed symptoms again including generalized pain, difficulty opening the mouth, inability to eat, edema in both lower limbs, and hypoproteinemia. Nutritional support was administered. Unfortunately, the patient passed away on October 21, 2024, with an overall survival (OS) of 23 months. The course timeline depicting the patient’s treatments was illustrated in **Figure 4**.

## Discussion

Duodenal adenocarcinoma is a rare malignancy, accounting for less than 1% of all gastrointestinal cancers (5). An international multicenter cohort study including 3,809 patients with five types of ampullary cancers, such as pancreatic ductal adenocarcinoma, distal bile duct cancer, duodenal adenocarcinoma, intestinal ampullary cancer, and pancreatobiliary ampullary cancer, found that duodenal adenocarcinoma shares oncological characteristics with intestinal-type ampullary cancer. Both have significantly better overall survival (OS) rates than other types of ampullary cancers (19). A separate multicenter retrospective study involving 2,189 patients indicated a 5-year OS rate of approximately 55.86% for patients undergoing radical surgery, with radical surgery correlating with longer OS (30). For stage IV patients, the 5-year survival rate is only 4.3%, with common metastatic sites including the peritoneum, liver, and lungs (31).

Given that the patient had developed resistance to both chemotherapy and immunotherapy, the multidisciplinary team actively considered whether targeted therapy could confer a survival benefit to the patient. No targeted agent has been approved specifically for KRAS p.G12R mutations. Sotorasib and adagrasib have been approved for use in NSCLC,which could be obtained through compassionate use. However, in the present case, the tumour harboured p.G12R (c.34G>C), which encodes arginine at codon 12 and lacks the sulfhydryl group required for drug engagement. Pre-clinical studies and early clinical data demonstrated primary resistance of p.G12R models to both sotorasib and adagrasib (32, 33). On this basis, the team elected not to pursue compassionate use.

Another striking feature of this case is the emergence of multifocal bone metastases only 4 months after an R0 pancreatoduodenectomy that had been followed by negative staging CT and normal serum CEA/CA-19-9. One plausible explanation is that occult micrometastases were already present at the time of surgery, with the peri-ampullary carcinoma having seeded early through the rich peri-duodenal venous plexus. Although the post-operative CT showed no lytic or blastic changes, this may simply reflect that the tumour deposits were below the spatial-resolution threshold of CT. Another factor could be the inadequate intensity of initial systemic therapy. Because of low body weight, poor performance status, and the need to await drain removal, this patient received only a single cycle of capecitabine monotherapy during the first five post-operative weeks. These observations suggest that high-risk ampullary carcinomas (those with lymphovascular invasion, T3 stage, or node positivity) should be considered for baseline staging with whole-body bone SPECT or PET-CT, and that early, appropriately intensified systemic treatment should be planned whenever feasible.

The abscopal effect is a notable phenomenon increasingly reported in recent years, induced by various local treatment modalities, particularly when combined with immune checkpoint inhibitors (17, 18, 27, 28). Radiotherapy is one such treatment known to induce the abscopal effect in cancers such as esophageal cancer (34), gastric cancer (35), and non-small cell lung cancer (36). Irradiation induces various changes in tumor cells and the tumor microenvironment, including the release of damage-associated molecular patterns (DAMPs) and tumor-associated antigens (TAAs), the accumulation of cytoplasmic DNA, the upregulation of signals that promote the recruitment of dendritic cells (DCs), and the expression of genes involved in immune regulation and cellular stress responses (37, 38). These changes enhance the antigenicity of tumor cells, activate immune signaling pathways such as cGAS-STING (39), and promote the recruitment of antitumor effector T cells. This is referred to as the *in situ* vaccination phenomenon, where antigen-presenting cells (APCs) recognize tumor antigens and present them to cytotoxic T cells, which can then target both primary and distant tumors (40). This may be one of the mechanisms underlying the abscopal effect.

Several biological factors influence the occurrence of the abscopal effect, including immune cells, cytokines, tumor-draining lymph nodes (TDLNs), and factors such as p53 status, mitochondria, and exosomes that may impact the antitumor immune response (26). Among immune cells, T cells that target endogenous tumor antigens are crucial to antitumor function (41, 42). In colorectal cancer patients, those with low levels of CD8+ T cell infiltration have significantly shorter OS compared to those with high levels (P = 0.01) (43), and the absence of CD8+ T cell infiltration contributes to immune resistance (44). Studies also show that CD8+ T cells are integral to the abscopal effect. Research by Lai demonstrated that in highly immunogenic tumors, radiotherapy inhibits tumor growth and induces the abscopal effect, whereas this phenomenon does not occur in low immunogenic tumors. Depletion of CD8+ T cells using specific antibodies abrogates the abscopal effect (45). Additionally, the number of CD8+ T cells secreting tumor-specific IFN-γ correlates with the regression of tumors outside the radiation field (46).

In our case, the patient underwent multiple cycles of chemotherapy and immunotherapy, with a lymphocyte count of 1.34 × 10^9/L prior to radiotherapy. The total peripheral-blood lymphocyte count fell abruptly (0.1-0.3×10^9/L, 10-20% of baseline) during radiotherapy from 7 June to 18 July 2024. T-cell subset analysis showed that the absolute CD8+ T cell count remained > 100 cells/µL (nadir 143 cells/µL) and their fraction rose from 34% to 64%, coinciding with the appearance of an abscopal response. This suggests that an early abscopal effect can still occur despite severe radiation-induced lymphopenia, provided a critical number of CD8+ T cells are preserved. In contrast, total B cells and NK cells dropped to < 10 cells/µL, indicating that antibody-dependent or NK-cell–mediated mechanisms contributed little to the observed systemic effect.However, after the radiotherapy course, the maxillofacial lesion started to enlarge. These phenomena may be attributed to treatment interruption and the patient’s poor physical status. Unfortunately, serial T-cell subset data during radiotherapy were not available, and this absence limits our ability to definitively link temporal immune dynamics to the observed abscopal response.

Pro-inflammatory cytokines, such as interleukin-6 (IL-6), play a significant role in the occurrence, development, and metastasis of tumors (47). IL-6 is involved in immune responses, inflammatory reactions, and various physiological processes. IL-6 has dual roles in tumors, with chronic activation promoting tumor progression and acute activation having anti-tumor effects (48). Acute IL-6 activation stimulates T cell activation, proliferation, and increased infiltration of CD8+ T cells, enhancing anti-tumor adaptive immunity, tumor cell apoptosis, and inhibiting tumor growth (48). In pancreatic cancer, IL-6 is critical for TGFβ-specific T cells to exert anti-tumor effects (49). In colorectal cancer, high IL-6 levels correlate with poor prognosis, larger tumor lesions, and liver metastasis (50, 51). Experimental studies suggest that blocking IL-6 can enhance CD8+ T cell accumulation and increase tumor cell sensitivity to anti-PD-1 treatment (47).

In our case, IL-6 levels remained elevated throughout treatment. During radiotherapy, IL-6 levels fluctuated mildly, peaking at 17.8 pg/mL at the end of treatment. Afterward, IL-6 levels began to decrease. The levels of IL-6 appeared closely associated with the patient’s physical status, radiotherapy, and tumor control. When the patient was in better condition, with pain relief and an abscopal effect, IL-6 levels were lower. However, after radiotherapy, when the patient’s physical condition worsened, the abscopal effect did not reappear, consistent with previous findings (47, 52). Regrettably, we did not monitor IL-6 levels at the outset of radiotherapy, limiting the ability to perform a more detailed comparison alongside imaging results during the abscopal effect.

This patient with duodenal adenocarcinoma developed multiple bone and soft-tissue metastases after several lines of therapy. Serial serum CA19-9, CA-125 and IL-6 levels tracked closely with radiologic tumour burden, underscoring the value of these markers as indicators of disease activity. When PET-CT demonstrated extensive progression in December 2023, CA19–9 and CA-125 rose concomitantly to 68.6 U/mL and 78.9 U/mL, respectively. After switching to irinotecan plus raltitrexed and bevacizumab, marker levels fell, suggesting transient cytoreductive activity. Re-enlargement of the right piriformis lesion in February 2024, accompanied by renewed increases in CA19-9 (99.9 U/mL) and CA-125 (121 U/mL), signalled secondary resistance—consistent with prior results of median progression-free survival <4 months for irinotecan–bevacizumab regimens (53). Following palliative radiotherapy to body metastasis on 7 June 2024, pain resolved; the irradiated rib lesion regressed, an unirradiated right maxillofacial focus shrank markedly, and CEA, CA-125 and CA19–9 declined rapidly (**Figure 1**). This systemic response likely reflects radiation-induced immunogenic cell death that released additional tumour antigens and transiently reactivated systemic anti-tumour immunity. Unfortunately, markers rebounded after irradiation, paralleling rapid performance-status decline and inability to resume chemotherapy. These observations suggest that local radiotherapy combined with immunotherapy may elicit an abscopal effect, providing short-term benefit and a therapeutic window in advanced duodenal adenocarcinoma. Future trials should systematically optimise radiation dose, fractionation and immunotherapy maintenance to prolong abscopal responses and improve long-term outcomes.

Other factors, such as radiation dose, fractionation, and irradiation type, also affect the abscopal effect (26). The effect is more commonly observed when hypofractionated radiotherapy is combined with immunotherapy (54, 55). Dewan’s research found that the abscopal effect was seen in mice treated with anti-CTLA-4 antibody combined with fractionated radiotherapy (8 Gy × 3 fractions or 6 Gy × 5 fractions) but not with single-fraction radiotherapy (46). While the abscopal effect induced solely by radiotherapy is rare (56), previous studies have documented its occurrence in mouse models of colorectal cancer, Lewis lung carcinoma (LLC), and fibrosarcoma (57, 58). In this case, the patient received conventional fractionated radiotherapy (38Gy/19f, 5 fractions per week). After surgery, the patient underwent chemotherapy and immunotherapy, but upon encountering drug resistance and disease progression, she received palliative radiotherapy. No other systemic medications were administered during radiotherapy. This case demonstrates that the abscopal effect can be induced solely by radiotherapy. The patient, with advanced cancer and multiple metastases, received radiotherapy targeting 8 lesions. The substantial tumor burden after irradiation may have exposed various tumor antigens, activating the immune response and contributing to the abscopal effect.

## Conclusion

To our knowledge, this is the first case demonstrating the abscopal effect of radiotherapy in a late-stage duodenal cancer patient resistant to chemotherapy and immunotherapy. Radiotherapy controlled the primary lesion, provided palliative pain relief, and induced an abscopal effect by enhancing immune system response to distant metastatic lesions. For patients with multiple metastases whose dominant need is relief of tumour-related pain, localized radiotherapy remains a valid and effective palliative option. Given that this report is limited to a single patient, further work should be undertaken in the future to verify this phenomenon in other metastatic lesions within the same clinical context.

## Data Availability

The original contributions presented in the study are included in the article/**Supplementary Material**. Further inquiries can be directed to the corresponding authors.

## Supplementary material

Supplementary Figure 1 **(A, B)** Contrast-enhanced CT, 2022-11-30; **(C, D)** DWI sequence of MRCP, 2022-12-2; **(E, F)** T2-FLAIR sequence of MRCP; **(G, H)** Contrast-enhanced T1-weighted sequence of MRCP; **(I)** Coronal contrast-enhanced T1-weighted sequence of MRCP. Red arrows indicate the primary lesion; yellow arrows indicate metastatic lymph nodes.

Supplementary Figure 2 Relative positions of the eight lesions that received radiotherapy.

## References

[1] Siegel RL Miller KD Wagle NS Jemal A . Cancer statistics, 2023. CA Cancer J Clin. (2023) 73:17–48. doi: 10.3322/caac.21763, PMID: 36633525

[2] Bilimoria KY Bentrem DJ Wayne JD Ko CY Bennett CL Talamonti MS . Small bowel cancer in the United States: changes in epidemiology, treatment, and survival over the last 20 years. Ann Surg. (2009) 249:63–71. doi: 10.1097/SLA.0b013e31818e4641, PMID: 19106677

[3] Goldner B Stabile BE . Duodenal adenocarcinoma: why the extreme rarity of duodenal bulb primary tumors? Am Surg. (2014) 80:956–9. doi: 10.1177/000313481408001010, PMID: 25264638

[4] Ross RK Hartnett NM Bernstein L Henderson BE . Epidemiology of adenocarcinomas of the small intestine: is bile a small bowel carcinogen? Br J Cancer. (1991) 63:143–5. doi: 10.1038/bjc.1991.29, PMID: 1989654 PMC1971637

[5] Overman MJ Hu CY Kopetz S Abbruzzese JL Wolff RA Chang GJ . A population-based comparison of adenocarcinoma of the large and small intestine: insights into a rare disease. Ann Surg Oncol. (2012) 19:1439–45. doi: 10.1245/s10434-011-2173-6, PMID: 22187121 PMC3342860

[6] Cloyd JM George E Visser BC . Duodenal adenocarcinoma: Advances in diagnosis and surgical management. World J Gastrointest Surg. (2016) 8:212–21. doi: 10.4240/wjgs.v8.i3.212, PMID: 27022448 PMC4807322

[7] Yeo CJ Sohn TA Cameron JL Hruban RH Lillemoe KD Pitt HA . Periampullary adenocarcinoma: analysis of 5-year survivors. Ann Surg. (1998) 227:821–31. doi: 10.1097/00000658-199806000-00005, PMID: 9637545 PMC1191384

[8] Nandy K Patel D Kaderi ASA Deshpande G Ostwal V Ramaswamy A . Long-term outcomes after resection of extra-ampullary duodenal adenocarcinomas: single-center experience. J Gastrointest Surg. (2024) 28:1805–11. doi: 10.1016/j.gassur.2024.08.017, PMID: 39181233

[9] Kato T Ono Y Oba A Sato T Ito H Inoue Y . Comparison of the clinical efficacy of a new prognostic stratification for duodenal adenocarcinoma with that of TNM staging: The importance of T status with regard to the prognosis. Eur J Surg Oncol. (2023) 49:122–8. doi: 10.1016/j.ejso.2022.08.005, PMID: 35999143

[10] Wei X Chen K Li DC Li H Zhu L Wang ZG . Risk and prognostic factors for small bowel adenocarcinoma: A multicenter retrospective observational study in China. Clin Med Insights Oncol. (2022) 16:11795549221091207. doi: 10.1177/11795549221091207, PMID: 35496501 PMC9044781

[11] Wang X Zhang H XinZhang Liu Y . Abscopal effect: from a rare phenomenon to a new frontier in cancer therapy. biomark Res. (2024) 12:98. doi: 10.1186/s40364-024-00628-3, PMID: 39228005 PMC11373306

[12] Igarashi H Fukuda M Konno Y Takano H . Abscopal effect of radiation therapy after nivolumab monotherapy in a patient with oral mucosal melanoma: A case report. Oral Oncol. (2020) 108:104919. doi: 10.1016/j.oraloncology.2020.104919, PMID: 32713809

[13] D’Andrea MA Reddy GK . Extracranial systemic antitumor response through the abscopal effect induced by brain radiation in a patient with metastatic melanoma. Radiat Oncol J. (2019) 37:302–8. doi: 10.3857/roj.2019.00437, PMID: 31918469 PMC6952716

[14] Tsui JM Mihalcioiu C Cury FL . Abscopal effect in a stage IV melanoma patient who progressed on pembrolizumab. Cureus. (2018) 10:e2238. doi: 10.7759/cureus.2238, PMID: 29719740 PMC5922506

[15] Nam SW Han JY Kim JI Park SH Cho SH Han NI . Spontaneous regression of a large hepatocellular carcinoma with skull metastasis. J Gastroenterol Hepatol. (2005) 20:488–92. doi: 10.1111/j.1440-1746.2005.03243.x, PMID: 15740500

[16] Okuma K Yamashita H Niibe Y Hayakawa K Nakagawa K . Abscopal effect of radiation on lung metastases of hepatocellular carcinoma: a case report. J Med Case Rep. (2011) 5:111. doi: 10.1186/1752-1947-5-111, PMID: 21418591 PMC3069951

[17] Golden EB Demaria S Schiff PB Chachoua A Formenti SC . An abscopal response to radiation and ipilimumab in a patient with metastatic non-small cell lung cancer. Cancer Immunol Res. (2013) 1:365–72. doi: 10.1158/2326-6066.CIR-13-0115, PMID: 24563870 PMC3930458

[18] Okamoto M Sato H Gao X Ohno T . Pembrolizumab after carbon ion radiation therapy for alveolar soft part sarcoma shows a remarkable abscopal effect: A case report. Adv Radiat Oncol. (2022) 7:100893. doi: 10.1016/j.adro.2021.100893, PMID: 35198839 PMC8841365

[19] Song M Yoon H Yoon H Lee HM Chae YJ Chang JE . Enhanced anticancer efficacy of photodynamic therapy in combination with immunotherapy. J Photochem Photobiol B. (2024) 261:113048. doi: 10.1016/j.jphotobiol.2024.113048, PMID: 39476746

[20] Collins VG Hutton D Hossain-Ibrahim K Joseph J Banerjee S . The abscopal effects of sonodynamic therapy in cancer. Br J Cancer. (2024) 132:409–20. doi: 10.1038/s41416-024-02898-y, PMID: 39537767 PMC11876350

[21] Cano-Mejia J Shukla A Ledezma DK Palmer E Villagra A Fernandes R . CpG-coated pRussian blue nanoparticles-based photothermal therapy combined with anti-CTLA-4 immune checkpoint blockade triggers a robust abscopal effect against neuroblastoma. Transl Oncol. (2020) 13:100823. doi: 10.1016/j.tranon.2020.100823, PMID: 32652470 PMC7348061

[22] Shao C Yang M Pan Y Xie D Chen B Ren S . Case report: abscopal effect of microwave ablation in a patient with advanced squamous NSCLC and resistance to immunotherapy. Front Immunol. (2021) 12:696749. doi: 10.3389/fimmu.2021.696749, PMID: 34413851 PMC8368438

[23] Wetterwald L Papadopoulos S Tsoumakidou G Boughdad S Ferraro D Koulouris P . Abscopal effect induced by cryoablation in a 55-year-old patient with metastatic dedifferentiated liposarcoma: a case report. Ann Transl Med. (2024) 12:94. doi: 10.21037/atm-23-1868, PMID: 39507450 PMC11534740

[24] Wu M Liu J Seery S Meng X Yue J . Cytoreductive nephrectomy promoted abscopal effect of camrelizumab combined with radiotherapy for metastatic renal cell carcinoma: A case report and review of the literature. Front Immunol. (2021) 12:646085. doi: 10.3389/fimmu.2021.646085, PMID: 34211459 PMC8239433

[25] Ma L Li Y Sakamoto Y Xie L Suzuki S Yoshida Y . Optimal radiation dose to induce an abscopal effect by combining carbon-ion radiotherapy and anti-CTLA4 antibody. Neoplasia. (2024) 60:101099. doi: 10.1016/j.neo.2024.101099, PMID: 39674115 PMC11699741

[26] Yu B Gao Y Li J Gao F Zhang J Li L . Killing two birds with one stone: Abscopal effect mechanism and its application prospect in radiotherapy. Crit Rev Oncol Hematol. (2024) 196:104325. doi: 10.1016/j.critrevonc.2024.104325, PMID: 38462151

[27] Hiniker SM Chen DS Knox SJ . Abscopal effect in a patient with melanoma. N Engl J Med. (2012) 366:2035. doi: 10.1056/NEJMc1203984, PMID: 22621637

[28] Stamell EF Wolchok JD Gnjatic S Lee NY Brownell I . The abscopal effect associated with a systemic anti-melanoma immune response. Int J Radiat Oncol Biol Phys. (2013) 85:293–5. doi: 10.1016/j.ijrobp.2012.03.017, PMID: 22560555 PMC3415596

[29] Lutz S Balboni T Jones J Lo S Petit J Rich SE . Palliative radiation therapy for bone metastases: Update of an ASTRO Evidence-Based Guideline. Pract Radiat Oncol. (2017) 7:4–12. doi: 10.1016/j.prro.2016.08.001, PMID: 27663933

[30] Xiao Q Wu X Yuan C Gu Z Tang X Meng F . Clinicopathologic features and surgery-related outcomes of duodenal adenocarcinoma: A multicenter retrospective study. Surgery. (2024) 176:1745–53. doi: 10.1016/j.surg.2024.08.007, PMID: 39261238

[31] Teufel A Meindl-Beinker NM Hosel P Gerken M Roig A Ebert MP . Characteristics and outcome of patients with small bowel adenocarcinoma (SBA). J Cancer Res Clin Oncol. (2023) 149:4579–90. doi: 10.1007/s00432-022-04344-z, PMID: 36163558 PMC10349691

[32] Ostrem JM Peters U Sos ML Wells JA Shokat KM . K-Ras(G12C) inhibitors allosterically control GTP affinity and effector interactions. Nature. (2013) 503:548–51. doi: 10.1038/nature12796, PMID: 24256730 PMC4274051

[33] Awad MM Liu S Rybkin II Arbour KC Dilly J Zhu VW . Acquired resistance to KRAS(G12C) inhibition in cancer. N Engl J Med. (2021) 384:2382–93. doi: 10.1056/NEJMoa2105281, PMID: 34161704 PMC8864540

[34] Hai T Liu J Lai J Zhou L . A good response to anti-PD-1 monoclonal antibody plus SBRT in a patient with PD-L1-negative recurrent advanced esophageal cancer: a long-term follow-up case report of a possible abscopal effect. Front Oncol. (2024) 14:1369035. doi: 10.3389/fonc.2024.1369035, PMID: 38993639 PMC11236593

[35] Bao Z Tang Q Chen H Zhang B Shi W Gu D . An abscopal effect in a gastric cancer patient treated with combined chemoimmunotherapy and palliative radiotherapy. Immunotherapy. (2022) 14:1429–35. doi: 10.2217/imt-2022-0041, PMID: 36537254

[36] Vilinovszki O Andratschke N Huellner M Curioni-Fontecedro A Kroeze SGC . True abscopal effect in a patient with metastatic non-small cell lung cancer. Radiat Oncol. (2021) 16:194. doi: 10.1186/s13014-021-01920-4, PMID: 34600561 PMC8487536

[37] Corro C Dutoit V Koessler T . Emerging trends for radio-immunotherapy in rectal cancer. Cancers (Basel). (2021) 13:1374. doi: 10.3390/cancers13061374, PMID: 33803620 PMC8003099

[38] Galluzzi L Buque A Kepp O Zitvogel L Kroemer G . Immunogenic cell death in cancer and infectious disease. Nat Rev Immunol. (2017) 17:97–111. doi: 10.1038/nri.2016.107, PMID: 27748397

[39] Deng L Liang H Xu M Yang X Burnette B Arina A . STING-dependent cytosolic DNA sensing promotes radiation-induced type I interferon-dependent antitumor immunity in immunogenic tumors. Immunity. (2014) 41:843–52. doi: 10.1016/j.immuni.2014.10.019, PMID: 25517616 PMC5155593

[40] Ali Mohammad S Hak A Pogu SV Rengan AK . Radiotherapy, photodynamic therapy, and cryoablation-induced abscopal effect: Challenges and future prospects. Cancer Innov. (2023) 2:323–45. doi: 10.1002/cai2.53, PMID: 38090387 PMC10686191

[41] Vanpouille-Box C Diamond JM Pilones KA Zavadil J Babb JS Formenti SC . TGFbeta is a master regulator of radiation therapy-induced antitumor immunity. Cancer Res. (2015) 75:2232–42. doi: 10.1158/0008-5472.CAN-14-3511, PMID: 25858148 PMC4522159

[42] Matsunaga A Ueda Y Yamada S Harada Y Shimada H Hasegawa M . Carbon-ion beam treatment induces systemic antitumor immunity against murine squamous cell carcinoma. Cancer. (2010) 116:3740–8. doi: 10.1002/cncr.25134, PMID: 20564091

[43] Funada Y Noguchi T Kikuchi R Takeno S Uchida Y Gabbert HE . Prognostic significance of CD8+ T cell and macrophage peritumoral infiltration in colorectal cancer. Oncol Rep. (2003) 10:309–13. doi: 10.3892/or.10.2.309, PMID: 12579264

[44] Sun R Limkin EJ Vakalopoulou M Dercle L Champiat S Han SR . A radiomics approach to assess tumour-infiltrating CD8 cells and response to anti-PD-1 or anti-PD-L1 immunotherapy: an imaging biomarker, retrospective multicohort study. Lancet Oncol. (2018) 19:1180–91. doi: 10.1016/S1470-2045(18)30413-3, PMID: 30120041

[45] Lai JZ Zhu YY Liu Y Zhou LL Hu L Chen L . Abscopal effects of local radiotherapy are dependent on tumor immunogenicity. Front Oncol. (2021) 11:690188. doi: 10.3389/fonc.2021.690188, PMID: 34249740 PMC8264447

[46] Dewan MZ Galloway AE Kawashima N Dewyngaert JK Babb JS Formenti SC . Fractionated but not single-dose radiotherapy induces an immune-mediated abscopal effect when combined with anti-CTLA-4 antibody. Clin Cancer Res. (2009) 15:5379–88. doi: 10.1158/1078-0432.CCR-09-0265, PMID: 19706802 PMC2746048

[47] Li W Wu Z Meng W Zhang C Cheng M Chen Y . Blockade of IL-6 inhibits tumor immune evasion and improves anti-PD-1 immunotherapy. Cytokine. (2022) 158:155976. doi: 10.1016/j.cyto.2022.155976, PMID: 35921790

[48] Orange ST Leslie J Ross M Mann DA Wackerhage H . The exercise IL-6 enigma in cancer. Trends Endocrinol Metab. (2023) 34:749–63. doi: 10.1016/j.tem.2023.08.001, PMID: 37633799

[49] Perez-Penco M Byrdal M Lara de la Torre L Ballester M Khan S Siersbaek M . The antitumor activity of TGFbeta-specific T cells is dependent on IL-6 signaling. Cell Mol Immunol. (2025) 22:111–26. doi: 10.1038/s41423-024-01238-7, PMID: 39653766 PMC11685413

[50] Chung YC Chang YF . Serum interleukin-6 levels reflect the disease status of colorectal cancer. J Surg Oncol. (2003) 83:222–6. doi: 10.1002/jso.10269, PMID: 12884234

[51] Groblewska M Mroczko B Wereszczynska-Siemiatkowska U Kedra B Lukaszewicz M Baniukiewicz A . Serum interleukin 6 (IL-6) and C-reactive protein (CRP) levels in colorectal adenoma and cancer patients. Clin Chem Lab Med. (2008) 46:1423–8. doi: 10.1515/CCLM.2008.278, PMID: 18844497

[52] Hu Y Zhou Y Du S Zhu W Chen Y Zeng Z . Effect of serum interleukin-6 concentration on the prognosis after radiotherapy for patients with hepatocellular carcinoma. Can J Gastroenterol Hepatol 2024. (2024) p:4696097. doi: 10.1155/cjgh/4696097, PMID: 39619013 PMC11606697

[53] Walter T Lievre A Coriat R Malka D Elhajbi F Di Fiore F . Bevacizumab plus FOLFIRI after failure of platinum-etoposide first-line chemotherapy in patients with advanced neuroendocrine carcinoma (PRODIGE 41-BEVANEC): a randomised, multicentre, non-comparative, open-label, phase 2 trial. Lancet Oncol. (2023) 24:297–306. doi: 10.1016/S1470-2045(23)00001-3, PMID: 36739879

[54] Vanpouille-Box C Alard A Aryankalayil MJ Sarfraz Y Diamond JM Schneider RJ . DNA exonuclease Trex1 regulates radiotherapy-induced tumour immunogenicity. Nat Commun. (2017) 8:15618. doi: 10.1038/ncomms15618, PMID: 28598415 PMC5472757

[55] Roger A Finet A Boru B Beauchet A Mazeron JJ Otzmeguine Y . Efficacy of combined hypo-fractionated radiotherapy and anti-PD-1 monotherapy in difficult-to-treat advanced melanoma patients. Oncoimmunology. (2018) 7:e1442166. doi: 10.1080/2162402X.2018.1442166, PMID: 30034949 PMC6053300

[56] Liu S Liao Y Chen Y Yang H Hu Y Chen Z . Effect of triple therapy with low-dose total body irradiation and hypo-fractionated radiation plus anti-programmed cell death protein 1 blockade on abscopal antitumor immune responses in breast cancer. Int Immunopharmacol. (2023) 117:110026. doi: 10.1016/j.intimp.2023.110026, PMID: 36934673

[57] Strigari L Mancuso M Ubertini V Soriani A Giardullo P Benassi M . Abscopal effect of radiation therapy: Interplay between radiation dose and p53 status. Int J Radiat Biol. (2014) 90:248–55. doi: 10.3109/09553002.2014.874608, PMID: 24350918

[58] Camphausen K Moses MA Menard C Sproull M Beecken WD Folkman J . Radiation abscopal antitumor effect is mediated through p53. Cancer Res. (2003) 63:1990–3., PMID: 12702593

