# Supplementary figures and images for: Case Report: Abscopal effect of radiotherapy in a patient with metastatic duodenal adenocarcinoma and resistance to chemoimmunotherapy
Source: Front Immunol. 2025 Dec 16;16:1643197. doi: 10.3389/fimmu.2025.1643197 (PMC12747931; doi:10.3389/fimmu.2025.1643197)

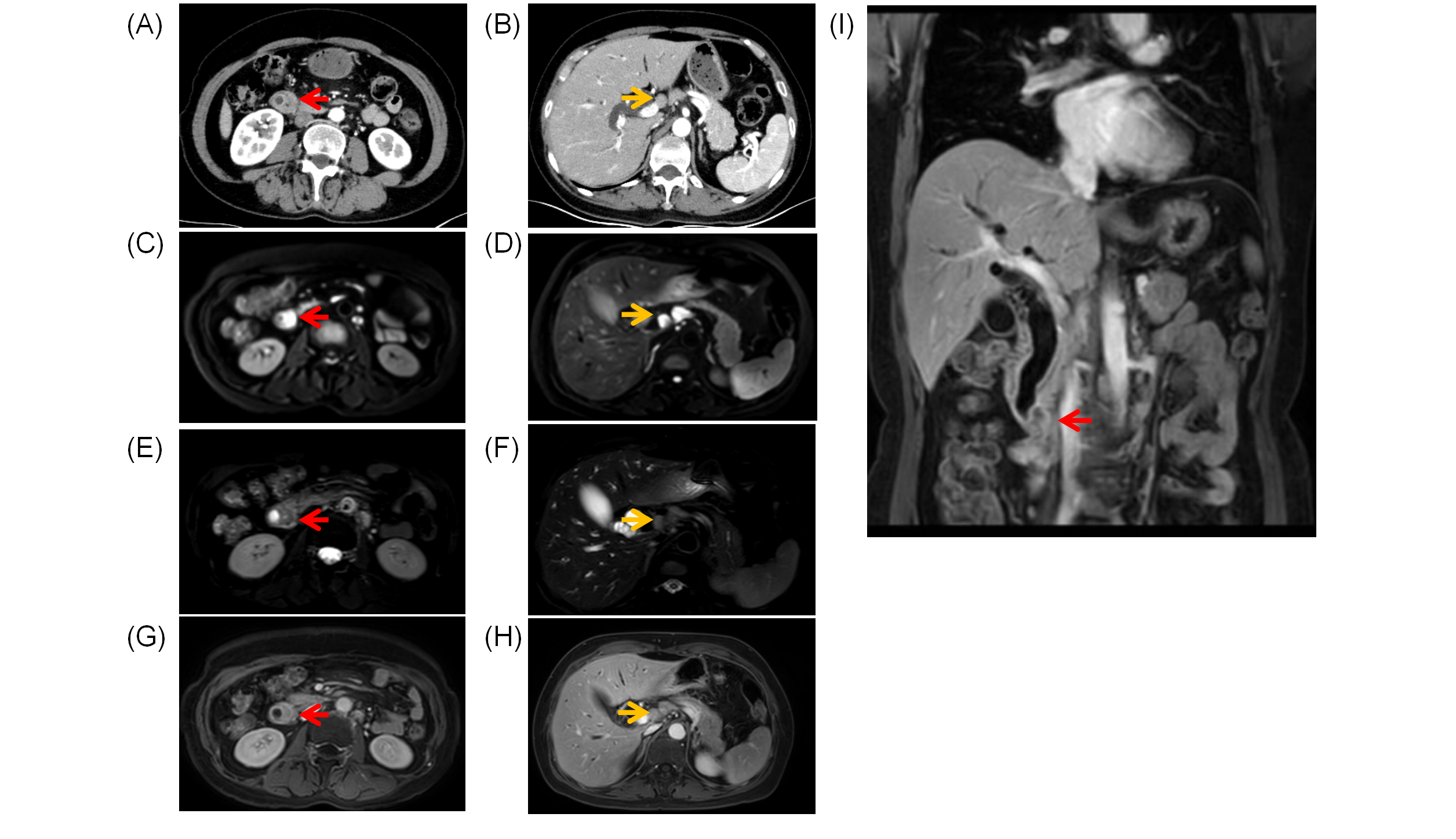

Supplement: Supplementary Figure 1 — (A, B) Contrast-enhanced CT, 2022-11-30; (C, D) DWI sequence of MRCP, 2022-12-2; (E, F) T2-FLAIR sequence of MRCP; (G, H) Contrast-enhanced T1-weighted sequence of MRCP; (I) Coronal contrast-enhanced T1-weighted sequence of MRCP. Red arrows indicate the primary lesion; yellow arrows indicate metastatic lymph nodes. [file Image1.tif]

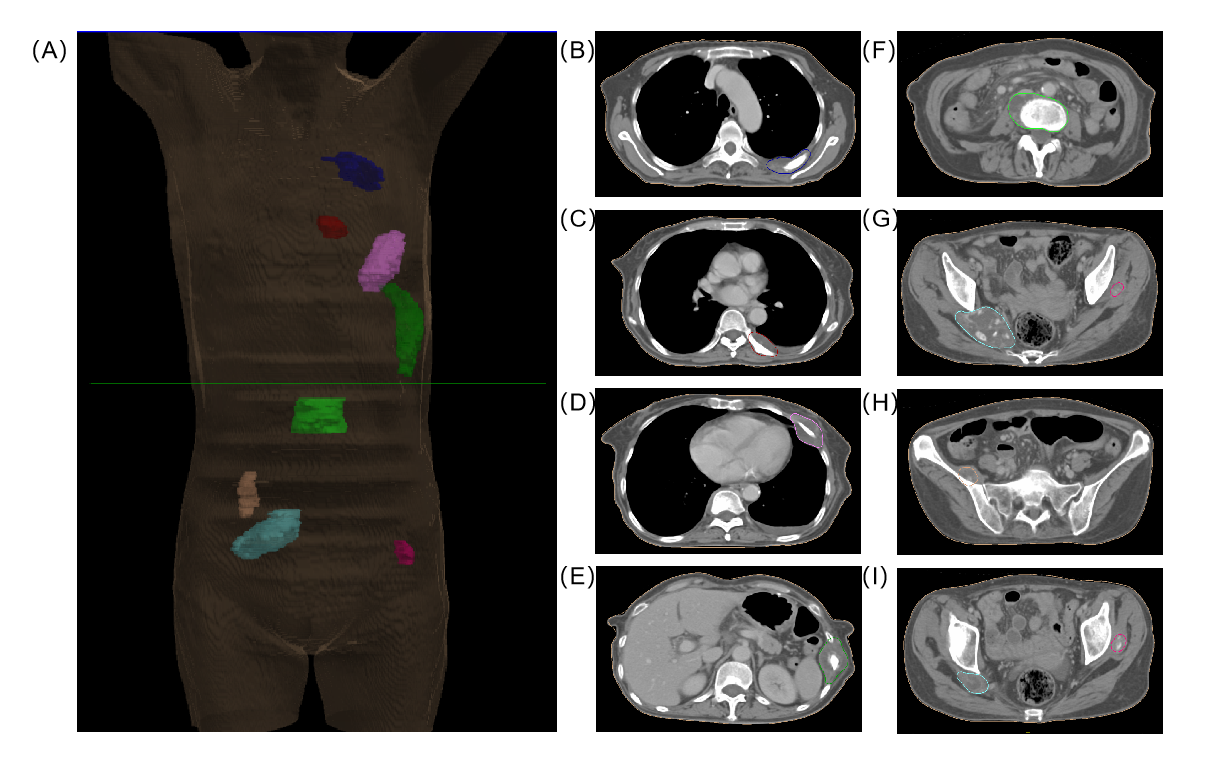

Supplement: Supplementary Figure 2 — Relative positions of the eight lesions that received radiotherapy. [file Image2.tif]
