# Supplementary material for: Case Report: Abscopal effect of radiotherapy in a patient with metastatic duodenal adenocarcinoma and resistance to chemoimmunotherapy
Source: Front Immunol. 2025 Dec 16;16:1643197. doi: 10.3389/fimmu.2025.1643197 (PMC12747931; doi:10.3389/fimmu.2025.1643197)
Supplement: Supplementary file 3 [file Table1.docx]

Supplementary Table 1.

Routine blood test results during the treatment period. The downward arrow indicates values below the normal lower limit.

| Date | Reference range | 2023/1/10 | 2023/4/19 | 2023/11/29 | 2024/1/12 | 2024/2/22 | 2024/6/3 | 2024/7/2 | 2024/7/8 | 2024/7/29 | 2024/8/15 | 2024/8/28 |
| --- | --- | --- | --- | --- | --- | --- | --- | --- | --- | --- | --- | --- |
| WBC | （3.50-9.50）*10^9/L | 4.64 | 4.13 | 4.1 | 5.31 | 4.94 | 6.55 | 1.78↓ | 1.02↓ | 2.27↓ | 3.4↓ | 3.09↓ |
| LY | （1.10-3.20）*10^9/L | 1.93 | 1.49 | 1.25 | 1.84 | 1.43 | 1.34 | 0.14↓ | 0.1↓ | 0.66↓ | 0.96↓ | 0.5↓ |
| NE | （1.80-6.30）*10^9/L | 2.37 | 2.17 | 2.52 | 3.06 | 3.06 | 4.85 | 1.49↓ | 0.67↓ | 1.1↓ | 1.94 | 2.16 |
| Hb | （115-150）g/L | 103↓ | 109↓ | 122 | 106↓ | 111↓ | 107↓ | 114↓ | 120 | 97↓ | 104↓ | 90↓ |
| PLT | （125-350）g/L | 328 | 288 | 270 | 470 | 291 | 350 | 104↓ | 141 | 178 | 227 | 128 |

Supplementary Table 2.

| Item | reference range | 2023/1/10 | 2023/4/19 | 2023/11/29 | 2024/1/12 | 2024/2/22 | 2024/6/3 | 2024/7/2 | 2024/7/29 | 2024/8/15 | 2024/8/28 |
| --- | --- | --- | --- | --- | --- | --- | --- | --- | --- | --- | --- |
| K+(mmol/L) | 3.50-5.30 | 5.2 | 4.5 | 5.2 | - | 4.4 | - | 4.6 | 4.98 | 3.53 | 4.58 |
| Na+(mmol/L) | 137.0-147.0 | 140.8 | 143.7 | 142.4 | - | 143.7 | - | 135.1 | 140.8 | 130 | 133 |
| Cl-(mmol/L) | 99.0-110.0 | 105 | 106 | 106 | - | 105 | - | 106 | 107 | 100 | 103 |
| HCO3-(mmol/L) | 22.0-29.0 | 29.1 | 30.3 | 28.6 | - | 30.2 | - | 20.2 | 21.4 | 19 | 22.6 |
| AG(mmol/L) | 8.00-16.00 | 11.9 | 11.9 | 13 | - | 12.9 | - | 13.5 | 17.38 | 14.53 | 11.98 |
| AST(U/L) | 13-35 | 36 | 59 | 39 | 36 | 36 | 26 | 30 | 17 | 22 | 17 |
| ALT(U/L) | 7-40 | 19 | 13 | 18 | 24 | 18 | 12 | 16 | 11 | 9 | 5 |
| ALP(U/L) | 50-135 | 173 | 224 | 139 | 156 | 200 | 207 | 135 | 97 | 114 | 106 |
| GGT(U/L) | 7-45 | 48 | 47 | 19 | 21 | 29 | 13 | 16 | 17 | 13 | 12 |
| TP(g/L) | 65.0-85.0 | 70 | 71 | 69 | 67 | 65 | 64 | 61 | 62.2 | 54.8 | 51.5 |
| ALB(g/L) | 40.0-55.0 | 40 | 43 | 42 | 38 | 39 | 36 | 37 | 35.6 | 29.4 | 30.1 |
| GLB(g/L) | 20.0-40.0 | 30 | 28 | 27 | 29 | 26 | 28 | 24 | 26.6 | 25.4 | 21.3 |
| A/G | 1.2-2.4 | 1.3 | 1.5 | 1.6 | 1.3 | 1.5 | 1.3 | 1.5 | 1.3 | 1.2 | 1.4 |
| CHE(U/L) | 5320-12920 | 5989 | 7157 | 7631 | 6476 | 7886 | 5421 | 5786 | 4926 | 3713 | 2346 |
| TBA(umol/L) | 0-10 | 9 | 13 | 45 | 37 | 51 | 20 | 13 | 5 | 6 | 10 |
| TBIL(umol/L) | 0.0-21.0 | 17 | 17 | 19 | 8 | 11 | 12 | 9 | 6.9 | 12.5 | 18.4 |
| DBIL(umol/L) | 0.0-8.0 | 11 | 6 | 6 | 3 | 5 | 6 | - | - | - | 7.2 |
| Urea(mmol/L) | 3.10-8.80 | 3 | 4.2 | 4.2 | 4.8 | 6.4 | 4.7 | 3.2 | 2.6 | 3.08 | 2.95 |
| Cr(umol/L) | 41-81 | 47 | 44 | 43 | 43 | 35 | 30 | 31 | 32 | 25 | 26 |
| Cys-C(mg/L) | 0.53-0.95 | 1.17 | 1.04 | 1.07 | 0.99 | 0.9 | 0.91 | 0.71 | 0.53 | 0.45 | 0.51 |
| eGFR | - | 103.5 | 105.8 | 105.8 | 105.8 | 113.2 | 119.1 | 117.8 | 116.6 | 125.6 | 124 |

The blood biochemistry test results during the treatment period.

Supplementary Table 3.

The lymphocyte classification count results during the treatment period.

| Item | reference range | 2024/5/6 | 2024/7/18 | 2024/8/21 |
| --- | --- | --- | --- | --- |
| CD4%(%) | (31-60) | 50 | 29 | 20 |
| CD4(cells/ul) | (410-1590) | 528 | 64 | 147 |
| CD8%(%) | (13-41) | 34 | 64 | 73 |
| CD8(cells/ul) | (190-1140) | 357 | 143 | 539 |
| CD4/CD8 |  | 1.48 | 0.45 | 0.27 |
| CD3%(T%)(%) | (55-84) | 85 | 95 | 96 |
| CD3(T)(cells/ul) | (690-2540) | 886 | 213 | 708 |
| CD16+56%(NK%)(%) | (5-27) | 11 | 3 | 3 |
| CD16+56(NK)(cells/ul) | (90-590) | 113 | 8 | 19 |
| CD19%(B%)(%) | (6-25) | 3 | <1 | <1 |
| CD19(B)(cells/ul) | (90-660) | 26 | <1 | <1 |
| Lymphs(CD45+)(cells/ul) | (1100-3200) | 1048 | 224 | 735 |

Supplementary Table 4.

The serum cytokines results during the treatment period.

| Date | reference range | 2024/5/6 | 2024/7/16 | 2024/7/18 | 2024/7/29 | 2024/8/15 | 2024/8/21 | 2024/8/23 | 2024/8/28 |
| --- | --- | --- | --- | --- | --- | --- | --- | --- | --- |
| IL-2(pg/mL) | ≤5.71 | <2.44 | <2.44 | <2.44 | <2.44 | <2.44 | <2.44 | <2.44 | <2.44 |
| IL-4(pg/mL) | ≤3.0 | <2.44 | <2.44 | <2.44 | <2.44 | <2.44 | <2.44 | <2.44 | <2.44 |
| IL-6(pg/mL) | ≤5.30 | 13.91↑ | 11.93↑ | 17.8↑ | 13.95↑ | 10.6↑ | 66.52↑ | 39.71↑ | 27.39↑ |
| IL-10(pg/mL) | ≤4.91 | 3.32 | <2.44 | <2.44 | 2.53 | <2.44 | 4.03 | 2.53 | 3.95 |
| IL-17A(pg/mL) | ≤20.60 | <10.00 | <10.00 | <10.00 | <10.00 | <10.00 | <10.00 | <10.00 | <10.00 |
| IFN-γ(pg/mL) | ≤7.42 | 3.13 | <2.44 | <2.44 | <2.44 | <2.44 | <2.44 | <2.44 | <2.44 |
| TNF-α(pg/mL) | ≤4.60 | <2.44 | <2.44 | <2.44 | <2.44 | <2.44 | <2.44 | <2.44 | <2.44 |
